# Aesop: A framework for developing and researching arts in health programmes

**DOI:** 10.1080/17533015.2014.924974

**Published:** 2014-07-03

**Authors:** Daisy Fancourt, Tim Joss

**Affiliations:** ^a^ Royal College of Music, Centre for Performance Science, Prince Consort Road, LondonSW7 2BS, UK; ^b^ Chelsea and Westminster Health Charity, Chelsea and Westminster Hospital NHS Foundation Trust, 4 Verney House, 1b Hollywood Road, London, SW10 9HS, UK; ^c^ The Rayne Foundation, 100 George Street, LondonW1U 8NU, UK

**Keywords:** methods, action research, experimental design, art forms, health issues

## Abstract

The field of arts in health is currently undergoing a burgeoning in activity. However, there remains a problem surrounding research into this field. Arts in health research can be confusing and is frequently misunderstood by those working in the arts and in health, artists, reviewers, researchers and funders. Aesop 1 is a framework specially devised to tackle these problems. It synthesises existing arts research methodologies, health research methodologies, health policy documents and reporting guidelines in order to guide projects right from the initial idea for an arts intervention, through the development and design of a research project, its delivery and its dissemination. This article outlines the rationale behind the framework and explains how it should be used, with the aim of facilitating the running of arts and health research projects and increasing their rigour and acceptance within both the arts and health communities.

## Introduction

The field of arts in health research is currently undergoing a burgeoning in activity. There are increasing numbers of research reports being published in journals ranging from the arts to medicine, nursing, rehabilitation, psychology, the arts therapies, neuroscience, biology and technology. There are a growing number of international conferences on the topic, including the yearly Global Alliance for Arts and Health conference, which began in 1989, the recent International Conference for Culture, Health and Wellbeing in England, June 2013, the International Arts and Health Conference in Australia, November 2013, and the International Association for Music and Medicine Conference in Canada, June 2014. And the contribution of the arts to health is now even being recognised by some governments, including the endorsing of the National Arts and Health Policy Framework by the Federal, State and Territory Ministers in Australia; the work of the US National Endowment for the Arts which is working with federal agencies and providing funding for arts projects and research; the involvement of the Finnish government departments for Health & Social Care and Education & Culture in creating projects to support the health of older adults through the arts, and the creation of the All Party Parliamentary Group on Arts and Health in the UK.

However, there remains a problem surrounding research in this field. Arts in health research can be confusing and is frequently misunderstood by those working in the arts and in health, artists, reviewers, researchers and funders. For example, for researchers, there seems to be a constant friction between selecting methods that fit the stringent requirements of health research and methods that adequately capture the true essence and impact of the art involved; a tension that more often ends in compromise than collaboration. For artists, scientific jargon can form a barrier to being able to develop robust, publishable research studies. And for funders, arts in health can fall between the humanities and sciences, with aims and methodologies that may be unfamiliar to the other.

The crux of this problem lies with the fact that there is currently no reference point for arts in health research; no standard for the development, design, delivery and dissemination of such research projects. At the same time, creating a specific arts and health research protocol risks branding arts in health research as “exceptionalism” and alienating people from both the arts fields and health fields.

Consequently, in June 2013, an international working group formed of leading artists, arts researchers, health researchers, policy-makers and funders was convened to discuss this issue with the aim of finding a way of bringing arts and health research more into the research mainstream. This working group recognised that arts-in-health research is not a case apart from other research projects either in the arts or health fields; it is not an exception to the guidelines and frameworks that already exist. However, at the same time, its position straddling two disciplines means that pre-existing frameworks often do not provide adequate guidance for arts-in-health researchers, particularly where researchers may come from one or other field rather than having an equal schooling in both. So over the ensuing six months, a new framework was devised that aimed to bring together all the relevant methods, protocols and guidelines for both arts research and health research and map out a clear and simple path that allow arts-in-health researchers to design projects that fit the requirements and expectations of both fields: Aesop 1: a framework for developing and researching arts in health programmes.

Aesop 1 tracks projects right from the initial idea for an arts intervention, through the development and design of a research project, its delivery and its dissemination. This article outlines the basis for the framework and how it can be used to maximum effect (see Figure 2 for the complete framework).

## Methodological Basis

The methodological basis for the Aesop 1 framework is a synthesis of existing arts research methodologies, health research methodologies, health policy documents and reporting guidelines. The overall concept and main stages of the framework are adapted from the Medical Research Council's (MRC) guidelines for “Developing and evaluating complex interventions” (Craig et al., 2008; Medical Research Council, 2000). Arts-in-health interventions are by definition complex medical interventions. Furthermore, the MRC guidelines recognise

the difficulty of standardising the design and delivery of the interventions, [the need for] sensitivity to features of the local context, the organisational and logistical difficulty of applying experimental methods to service or policy change, and the length and complexity of the causal chains linking intervention with outcome.

As such they reflect many of the important considerations in arts-in-health research. Crucially, the MRC guidance also recognises the need to help research funders to “understand the constraints on evaluation design and recognise appropriate methodological choices” (Craig et al., 2008, p. 6); echoing another objective of the Aesop 1 framework.

However, the MRC guidelines do not provide any bespoke advice or guidance on social or arts-based interventions, making them sometimes hard to apply in practice. Consequently, the Aesop 1 framework combines the MRC guidelines with a number of other concepts and frameworks that can offer more support to arts-in-health interventions. A key example of this is the Participatory Action Research method (Baum, MacDougall, & Smith, 2006). This follows very similar paths to the MRC guidance but with a particular focus on experiential learning and participatory activities, which lends itself strongly to arts interventions. It also incorporates the concept of “reflection” (denoted by the “R” arrows in the diagram), whereby researchers can take stock of the research and make alterations or amendments to the research design at important stages in the process. This echoes the importance of reflective practice in the social sciences, arts and humanities, and increasingly in research carried out by health professionals.

The Aesop 1 framework also creates space for a number of other epistemologies including ethnography, grounded theory, phenomenology and discourse analysis. These have been synthesised into the framework and form some of the categories and scales to encourage researchers to consider their relevance to a project. An effort has been made to represent paradigms including post-positivism, social constructivism, advocacy and participatory views, and pragmatism, all of which are felt to be important to arts and health research.

A final consideration is the Nesta “Standards of Evidence for Impact Investing” (Puttick & Ludlow, 2012). This framework provides scales for the assessment of the impact and social benefit of interventions to maximise their value. Its ethos is to use evaluation to inform the development of interventions and increase the capacity for the delivery of the intervention, tying it in directly with the aims of the Aesop 1 framework and the spirit of arts-in-health interventions.

A number of other methods and guidelines have also been incorporated into the Aesop 1 framework, and the design is also such that it can be used alongside other frameworks, methods and techniques as a way of enhancing the understanding and application of intervention design, research methods and project reporting and implementation.

## Framework Stages

Overall the framework is split into six stages (Figure 1). Moving clockwise from the top:

1. Stage 1 denotes the arts intervention itself, whether it is being developed or implemented.Figure 1 (*Continued*).

**Figure:**
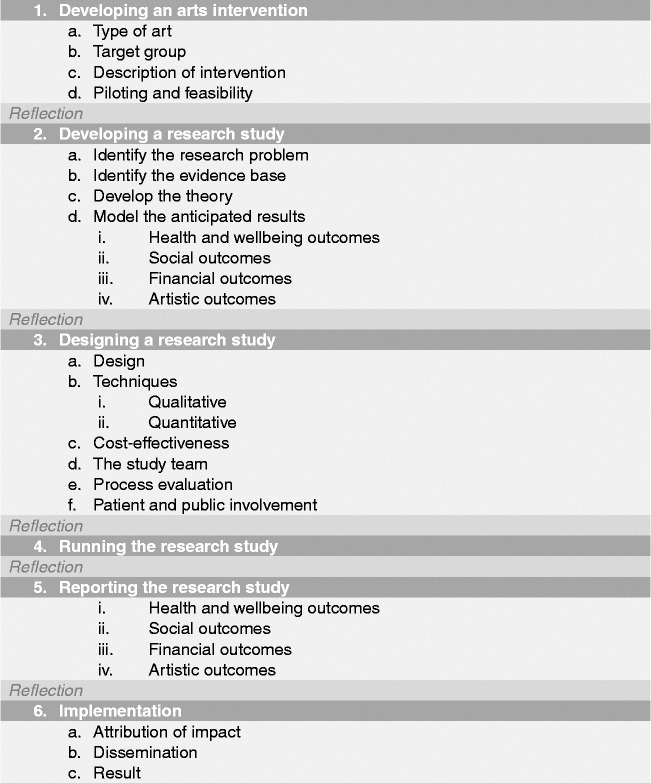

**Figure:**
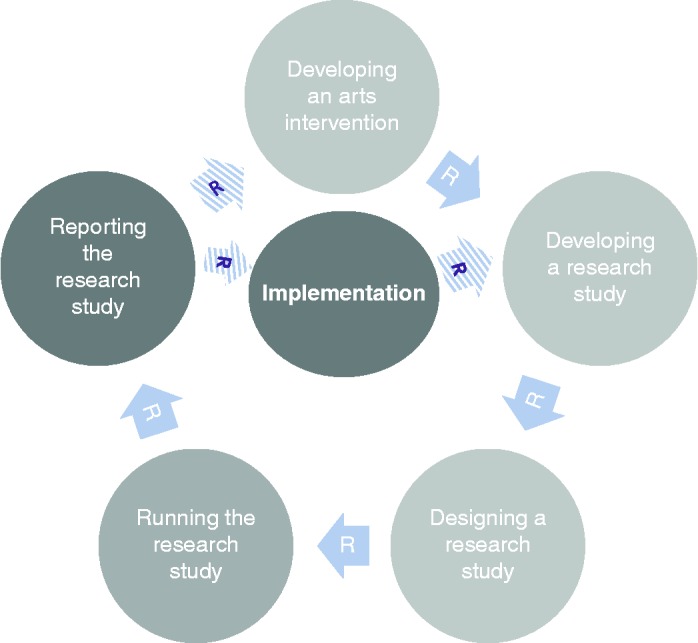
2. Stages 2 and 3 denote the development and design of the research study to investigate the effects of the arts intervention.
3. Stage 4 denotes the running of the research study.
4. Stages 5 denotes the analysis and dissemination of findings from the study. Following this, it is hoped that studies will result in the implementation of arts projects in healthcare (stage 6) and/or that, based on findings, future studies will then be designed and carried out (stages 2 and 3).

Each stage in the process involves a number of categories on a scale of 1–5, moving from “less comprehensive” to “more comprehensive”. Importantly, this should not be taken to mean that all studies are aiming to achieve a “5”, nor that they are underperforming if they only achieve a “1”. Indeed, it may not be appropriate or the intention for studies to attain the higher levels, and may not be of relevance to the parties involved or funders. Rather, this scale sets out the full spectrum of possibilities so that researchers have a clear awareness of the options available and can make an informed decision of where to situate themselves; how in-depth they want a research project to be and what impact they hope the study will have. These scales can also help research projects to plan how research projects will develop in the future a chart a trajectory from a small-scale pilot research project to a large multi-site trial. Overall, the aim is that increasing rigour and higher scores on the scales should lead to increasing acceptance of results from both the arts and health research communities (Figure 2).Figure 2 (*Continued*)

**Figure:**
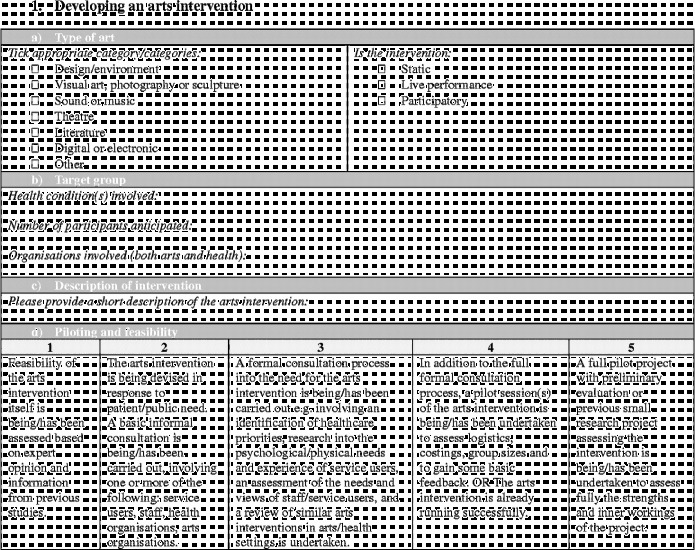

**Figure:**
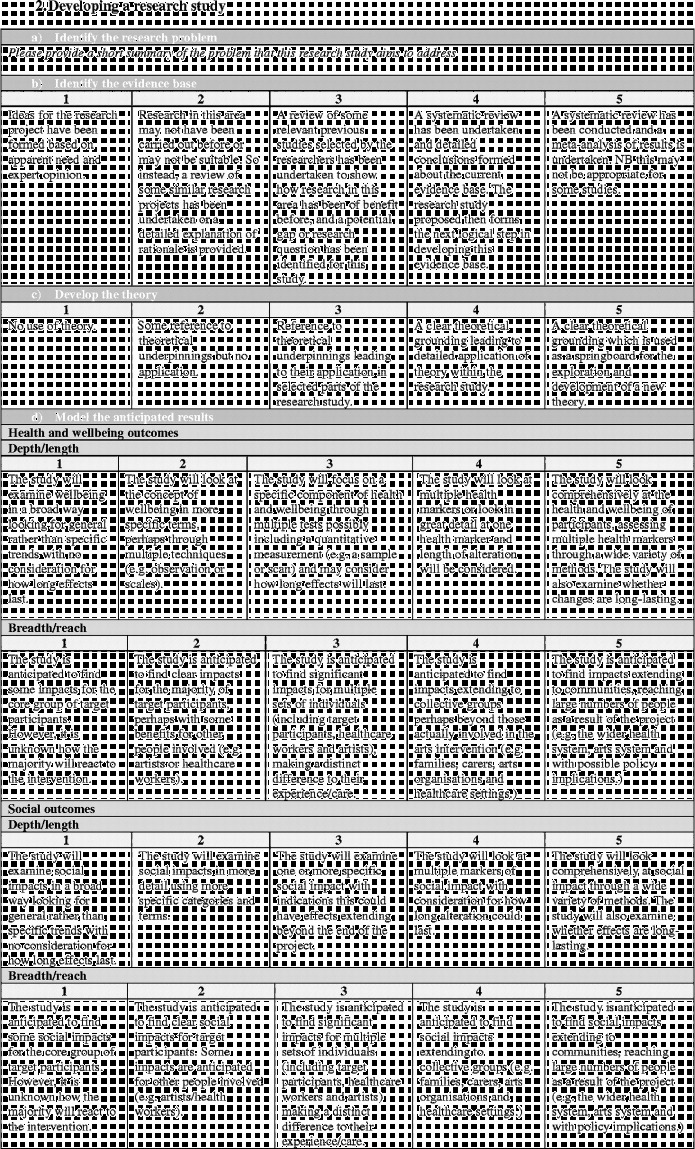

**Figure:**
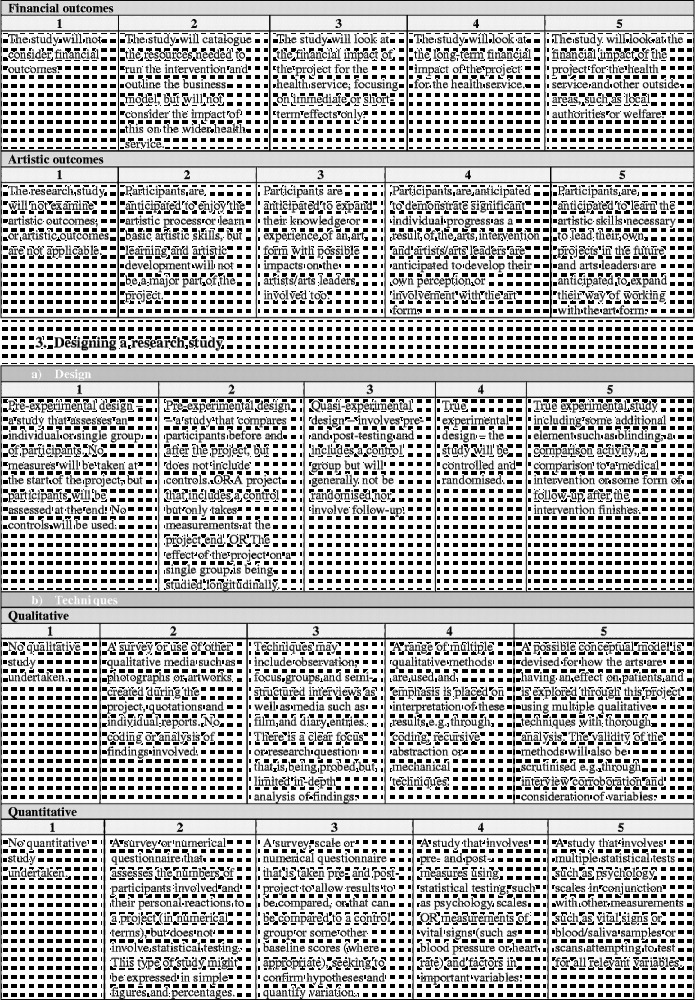

**Figure:**
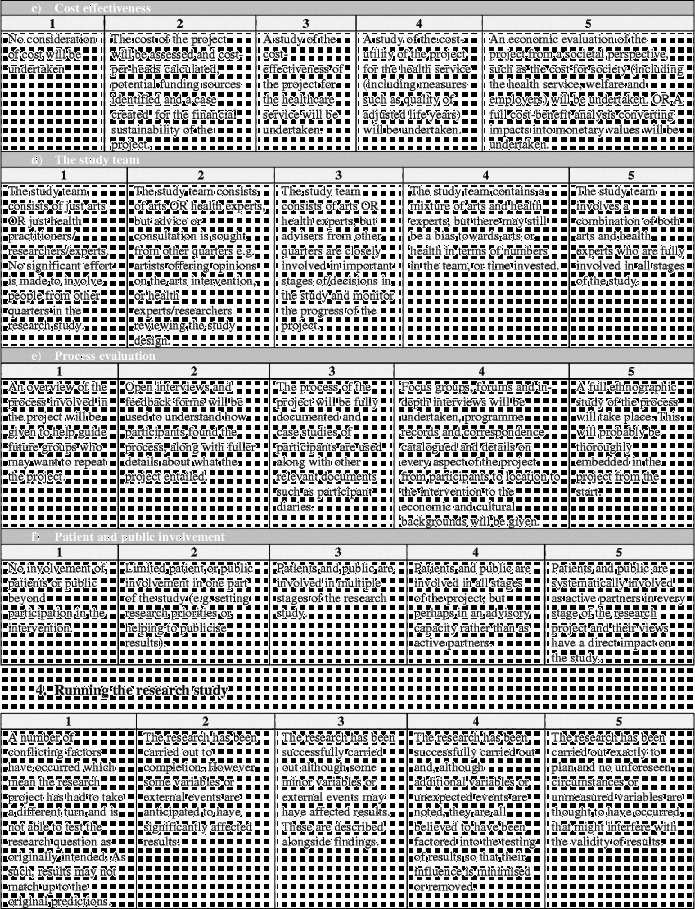

**Figure:**
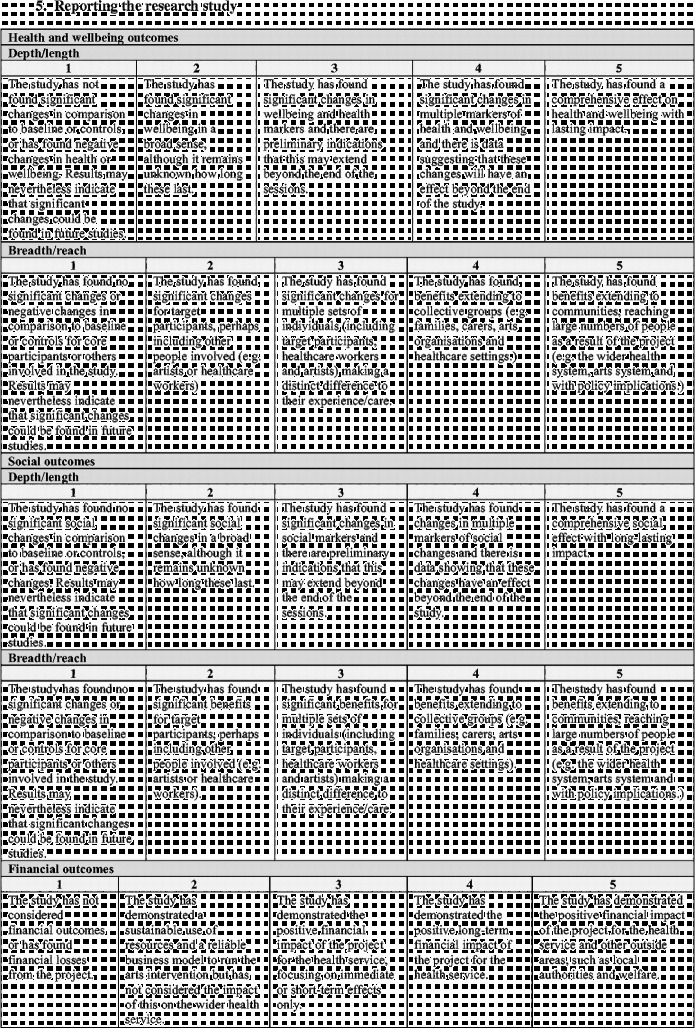

**Figure:**
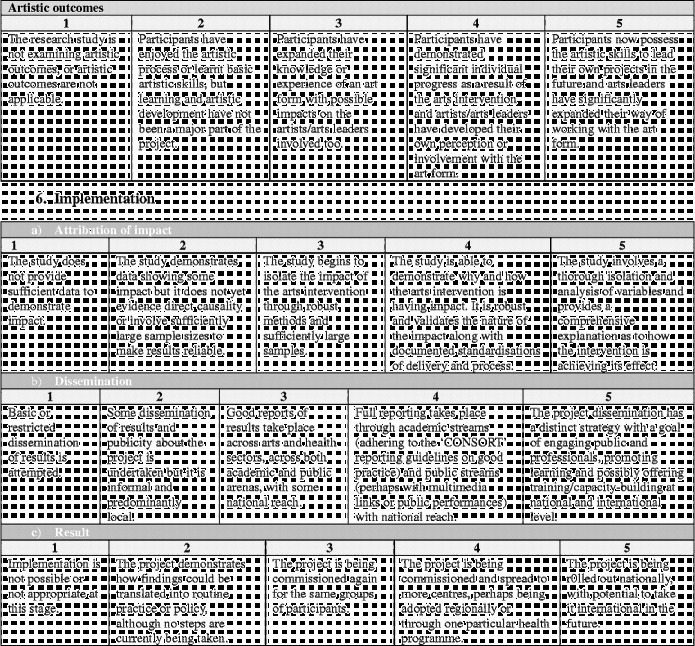


## Assessing Research Strength

Building on the scales used throughout the framework, it may also be beneficial to find the “score” for how comprehensive in its investigation a research project is overall (Figure 3). As with the scales, this is not to say that lower scoring research is inferior in status. Rather, such studies will demonstrate that the research questions being investigated are still in the early stages of being explored. However, it is hoped that this framework will allow researchers to position their study as a whole in a broad context of spectrums of design and research style and allow similar studies to be related to one another. As more studies are undertaken and published, it will hopefully be possible for the depth of the research question to be probed and more in-depth studies carried out.Figure 3 (*Continued*)

**Figure:**
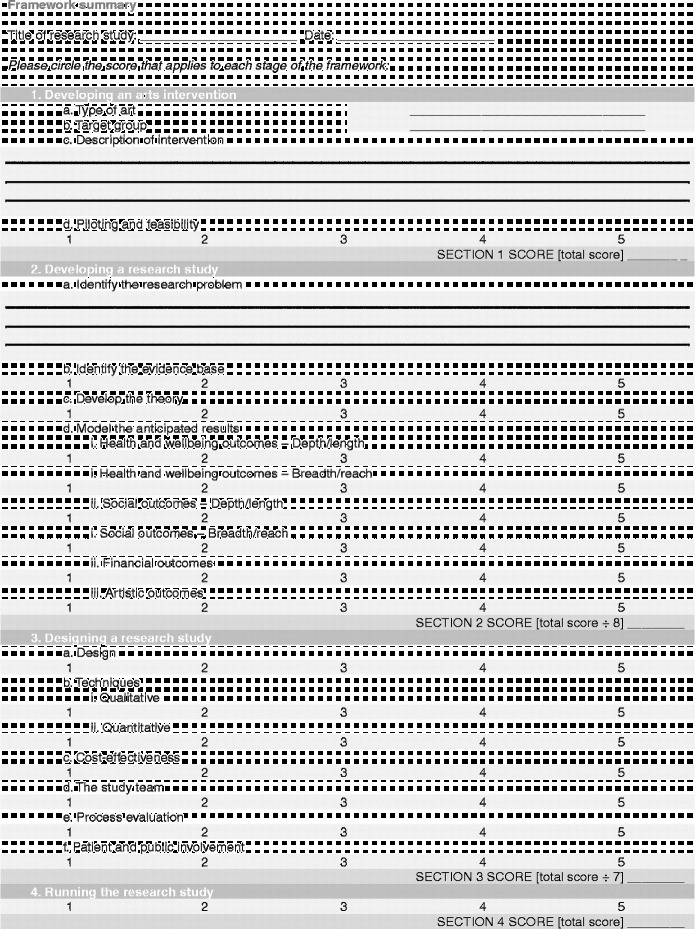

**Figure:**
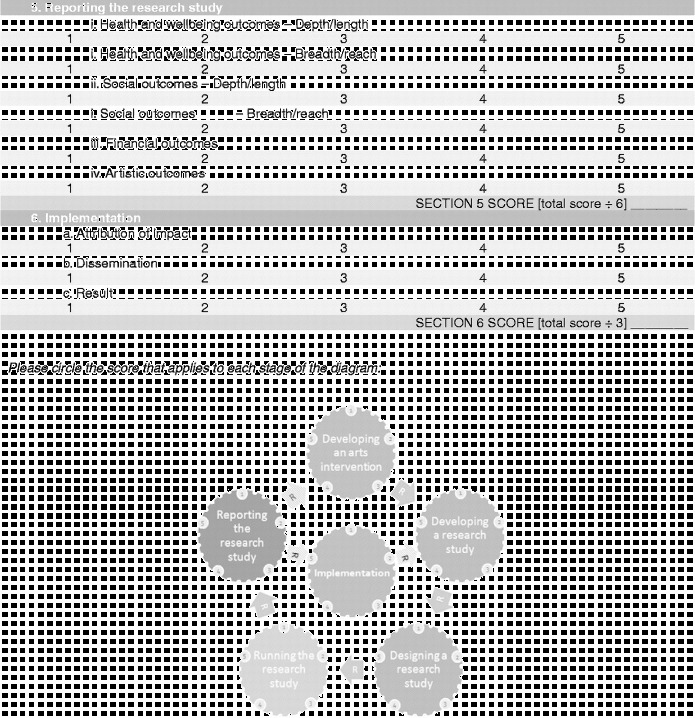


In order to calculate the research strength score, the framework ends with a “framework summary” document. This allows researchers to circle the scores from each of the sliding scales within the framework, calculate their score per section and then mark this on the diagram to give a visual representation of their research strength.
